# Prediction of Cerebral Amyloid With Common Information Obtained From Memory Clinic Practice

**DOI:** 10.3389/fnagi.2018.00309

**Published:** 2018-10-03

**Authors:** Jun Ho Lee, Min Soo Byun, Dahyun Yi, Bo Kyung Sohn, So Yeon Jeon, Younghwa Lee, Jun-Young Lee, Yu Kyeong Kim, Yun-Sang Lee, Dong Young Lee

**Affiliations:** ^1^Department of Neuropsychiatry, Seoul National University Hospital, Seoul, South Korea; ^2^Department of Clinical Pharmacology and Therapeutics, Seoul National University College of Medicine, Seoul, South Korea; ^3^Medical Research Center, Institute of Human Behavioral Medicine, Seoul National University, Seoul, South Korea; ^4^Department of Psychiatry, Sanggye Paik Hospital, Inje University College of Medicine, Seoul, South Korea; ^5^Department of Psychiatry and Behavioral Science, Seoul National University College of Medicine, Seoul, South Korea; ^6^Department of Neuropsychiatry, SMG-SNU Boramae Medical Center, Seoul, South Korea; ^7^Department of Nuclear Medicine, SMG-SNU Boramae Medical Center, Seoul, South Korea; ^8^Department of Nuclear Medicine, Seoul National University College of Medicine, Seoul, South Korea

**Keywords:** amyloid, prediction, Alzheimer’s disease, mild cognitive impairment, memory clinic

## Abstract

**Background:** Given the barriers prohibiting the broader utilization of amyloid imaging and high screening failure rate in clinical trials, an easily available and valid screening method for identifying cognitively impaired patients with cerebral amyloid deposition is needed. Therefore, we developed a prediction model for cerebral amyloid positivity in cognitively impaired patients using variables that are routinely obtained in memory clinics.

**Methods:** Six hundred and fifty two cognitively impaired subjects from the Korean Brain Aging Study for the Early diagnosis and prediction of Alzheimer disease (KBASE) and the Alzheimer’s Disease Neuroimaging Initiative-2 (ADNI-2) cohorts were included in this study (107 amnestic mild cognitive impairment (MCI) and 69 Alzheimer’s disease (AD) dementia patients for KBASE cohort, and 332 MCI and 144 AD dementia patients for ADNI-2 cohort). Using the cross-sectional dataset from the KBASE cohort, a multivariate stepwise logistic regression analysis was conducted to develop a cerebral amyloid prediction model using variables commonly obtained in memory clinics. For each participant, the logit value derived from the final model was calculated, and the probability for being amyloid positive, which was calculated from the logit value, was named the amyloid prediction index. The final model was validated using an independent dataset from the ADNI-2 cohort.

**Results:** The final model included age, sex, years of education, history of hypertension, apolipoprotein ε4 positivity, and score from a word list recall test. The model predicted that younger age, female sex, higher educational level, absence of hypertension history, presence of apolipoprotein ε4 allele, and lower score of word list recall test are associated with higher probability for being amyloid positive. The amyloid prediction index derived from the model was proven to be valid across the two cohorts. The area under the curve was 0.873 (95% confidence interval 0.815 to 0.918) for the KBASE cohort, and 0.808 (95% confidence interval = 0.769 to 0.842) for ADNI-2 cohort.

**Conclusion:** The amyloid prediction index, which was based on commonly available clinical information, can be useful for screening cognitively impaired individuals with a high probability of amyloid deposition in therapeutic trials for early Alzheimer’s disease as well as in clinical practice.

## Introduction

Cerebral amyloid beta (Aβ) deposition is the hallmark pathologic change in Alzheimer’s disease (AD). Since amyloid positron emission tomography (PET) imaging has made it possible to detect cerebral Aβ pathology in living human brains (Klunk et al., 2004), there has been a drastic paradigm shift in the diagnosis of AD, especially in clinical research including therapeutic clinical trials. Amyloid PET has been widely adopted in various clinical studies for the accurate diagnosis of AD. At present, typical AD clinical trials target the early stages of AD and specifically target patients with mild cognitive impairment (MCI) due to AD or mild AD dementia (McKhann et al., 2011; Sperling et al., 2011) that is confirmed by positive amyloid deposition on PET. However, due to the high cost, limited availability of PET machines, and concern about radiation hazards, application of amyloid PET imaging is still limited. Although cerebrospinal fluid (CSF) Aβ examination is an alternative to measuring cerebral Aβ pathology, there are possible contraindications, i.e., increased intracranial pressure, coagulopathy or current use of anticoagulant, to the technique. It also requires staffs with the adequate training and appropriate facilities (Herukka et al., 2017; Simonsen et al., 2017). In addition, a consensus on the application of CSF biomarkers in clinical practice is still necessary, since there exist differences in the detailed methods of application among countries, and even among sites (Lewczuk et al., 2006; Verwey et al., 2009).

The limitations of these techniques highlight the need for easily applicable and valid prediction methods to identify cognitively impaired individuals with a high probability of amyloid deposition. Given that about 30% of screening failures in recent early AD clinical trials stem from enrichment with amyloid (Coric et al., 2015; Sevigny et al., 2016; Wolz et al., 2016), such prediction method is desperately needed to facilitate the search for trial candidates. Although several studies previously reported prediction models that used multiple clinical variables, including neuropsychological, laboratory, and neuroimaging measures (Bahar-Fuchs et al., 2013; Haghighi et al., 2015), a small sample size and lack of external validation has restricted the value of the models. Recently, excellent blood biomarker-based prediction methods for amyloid accumulation based on novel analytical techniques were reported (Burnham et al., 2014; Haghighi et al., 2015; Ovod et al., 2017; Park et al., 2017; Nakamura et al., 2018). However, their availability in real clinical research or practice is still far from reality because of the further requirement for standardized analytical procedures, optimal cutoffs based on large-scale normative data, and the implementation of the assay systems.

Therefore, we developed a prediction model for cerebral amyloid positivity in cognitively impaired patients using variables that are routinely obtained in memory clinics. We intended to maximize the availability as well as validity of the model for use in current clinical settings. The model was first developed using the data from the Korean Brain Ageing Study for the Early diagnosis and prediction of Alzheimer’s disease (KBASE) cohort (Byun et al., 2017), and was then externally validated using an independent dataset from the Alzheimer’s Disease Neuroimaging Initiative 2 (ADNI-2) (Petersen et al., 2010).

## Materials and Methods

### Participants

KBASE is a prospective, longitudinal cohort study that has recruited participants with a wide age range (55–90 years) and varying cognitive status since 2014 (Byun et al., 2017). The study is aimed at searching for new AD biomarkers and investigating how multi-faceted lifetime experiences and bodily changes contribute to the brain changes or brain pathologies related to AD. The baseline data from the KBASE cohort were used as the development set, which included 107 amnestic MCI and 69 very mild or mild probable AD dementia patients, according to the National Institute on Aging–Alzheimer’s Association (NIA-AA) criteria (McKhann et al., 2011). The ADNI-2 dataset was used for the external validation of the developed model. The ADNI is a multisite longitudinal cohort study that was designed to investigate the trajectories of biomarkers across the entire AD spectrum, from normal aging to AD dementia (Petersen et al., 2010). For ADNI-2, the follow-up length was expanded, and the breadth of cognitive status was increased by adding patients with early MCI and subjective memory complaint (SMC) (Beckett et al., 2015). In addition, the depth of imaging biomarkers was enhanced by adding florbetapir PET for all participants (Jagust et al., 2015). The ADNI-2 dataset used in the current study included 332 MCI and 144 AD dementia patients, and the data were downloaded on September 2017 from the public website ^1^.

### Clinical, Neuropsychological, and Laboratory Assessments

All subjects from the KBASE and ADNI-2 cohorts underwent comprehensive clinical and neuropsychological assessments according to each study protocol (Beckett et al., 2015; Jagust et al., 2015; Byun et al., 2017). KBASE participants were assessed via a standardized clinical assessment protocol in which the Korean version of the Consortium to Establish a Registry for Alzheimer’s Disease (CERAD-K) clinical assessment was incorporated (Lee et al., 2002). The protocol included various assessment tools for the evaluation of clinical diagnosis, severity, activities of daily living, depression status, current or past medical comorbidities, use of medication, and a vast amount of information on lifestyle factors. For the acquisition of accurate information, reliable informants were interviewed and medical records were reviewed. KBASE neuropsychological assessments were performed according to the standardized protocol, which incorporated the CERAD-K neuropsychological battery (Lee et al., 2004). ADNI-2 subjects also underwent thorough clinical and neuropsychological assessments as per the protocol (Beckett et al., 2015). The clinical assessment protocol included items similar to those of KBASE. For the neuropsychological assessment, a standardized protocol that incorporated 13 items from the Cognitive Subscale of the Alzheimer’s Disease Assessment Scale (ADAS-Cog) was used (Beckett et al., 2015). The subjects of both cohorts also underwent routine laboratory assessments that are part of memory clinic evaluations, including apolipoprotein E (APOE) genotyping.

### Neuroimaging

For the brain imaging, KBASE and ADNI-2 participants underwent amyloid PET and brain magnetic resonance imaging (MRI) at screening or baseline visit, according to each study protocol (Jack et al., 2015a; Jagust et al., 2015; Byun et al., 2017). KBASE participants underwent simultaneous three-dimensional Pittsburgh compound B (PiB) PET (PiB-PET) and 3D T1-weighted MR imaging using a 3.0T Biograph mMR scanner (Siemens, Washington, DC, United States), according to the manufacturer’s guidelines. For the KBASE cohort, amyloid positivity was determined based on PiB-PET. To measure the cerebral amyloid burden, a global cortical region of interest (ROI) consisting of the frontal, lateral parietal, posterior cingulate and precuneus, and lateral temporal regions was defined. A global PiB retention value, otherwise known as a standardized uptake value ratio (SUVR), was generated by dividing the mean value for all voxels within the ROI by the mean cerebellar uptake value (Choe et al., 2014). Each participant was classified as amyloid positive if the global SUVR value was >1.40 (Murray et al., 2015). To obtain structural brain MRI measures, T1-weighted MR images used in this study were automatically segmented using FreeSurfer version 5.3^2^, and the FreeSurfer segmentation outputs were visually inspected for each participant and manual correction was undertaken if needed for minor segmentation errors. Left and right hippocampal volumes were extracted and then added to yield the total hippocampal volume. The percentage ratio of the total hippocampal volume to the estimated total intracranial volume [i.e., hippocampal volume ratio (HVR)] was calculated and used as an index for neurodegeneration. For the ADNI-2 cohort, ^18^F-florbetapir PET was applied to each participant to define amyloid positivity. The global SUVR was the mean florbetapir uptake from the gray matter within the lateral and medial frontal, anterior and posterior cingulate, lateral parietal, and lateral temporal regions relative to uptake in the whole cerebellum, and this summary measure was used as the florbetapir cortical mean for each subject (Landau et al., 2012). Each subject was classified as amyloid positive if the global SUVR value was >1.10 (Joshi et al., 2012).

### Statistical Analysis

We developed the amyloid prediction model with the KBASE dataset using stepwise multivariate logistic regression analysis. Of the extensive variables from the KBASE dataset (Byun et al., 2017), only the variables that are routinely obtained in memory clinic practice were selected for the development of the prediction model. The selected clinical variables were age, sex, years of education, history of vascular risk factors such as hypertension, diabetes, or hyperlipidemia, cognitive diagnosis (MCI vs. AD dementia), and sum of the box score for clinical dementia rating (CDR-SB). The raw score of four neuropsychological tests, including the Word List Recall test (WLR), 15-item Boston Naming Test (BNT), Semantic Fluency test (SF), and Constructional Praxis test (CP), included in the CERAD neuropsychological battery were also selected (Lee et al., 2004). The selected tests are widely used and cover the main cognitive domains affected by AD: WLR for episodic memory, BNT for language, SF for executive function, and CP for visuospatial function. The results of the clinical laboratory assessments that are usually performed in memory clinic practice were also used, including thyroid function tests (thyroid stimulating hormone and free thyroxine), liver function tests (aspartate transaminase, alanine transaminase, alkaline phosphatase, and total bilirubin), renal panel (creatinine and blood urea nitrogen), complete blood cell count (white blood cell, red blood cell, hemoglobin, and platelet), vitamin B12, folate, glucose, total cholesterol, triglyceride, albumin, total protein, total calcium, phosphorus, uric acid, erythrocyte sedimentation rate, and APOE genotype. For the APOE genotyping, subjects with at least one ε4 allele were classified as APOE4 positive, and those without a ε4 allele were classified as APOE4 negative. Lastly, HVR was selected as a structural imaging variable.

The final model for amyloid positivity prediction (amyloid prediction model) was developed using a multivariate stepwise logistic regression analysis. Demographic variables including age, sex, and years of education were entered as fixed variables and all other variables were sequentially entered into the model using the forward likelihood ratio (LR) method. The logit value derived from the final model for each subject was calculated, and the probability for being amyloid positive, which was calculated from the logit value, was named the amyloid prediction index (API). Using API, receiver operating characteristic (ROC) curve analysis was performed and the area under the curve (AUC) value was calculated for the ROC curve. As the optimal cutoff point for API can vary according to the purpose and setting of its use, i.e., screening candidates for clinical trial or screening patients who need further evaluation using amyloid PET in memory clinic practice, we explored how the sensitivity, specificity, positive predictive value (PPV), negative predictive value (NPV), and accuracy value change as the API increases from 0.1 to 0.9 by 0.1 unit, instead of determining a single optimal cutoff value for API.

The final model was validated externally using the ADNI-2 dataset. From the ADNI-2 dataset, identical or nearly identical variables to the variables included in the final model were selected. Using the variables, the API score for each ADNI-2 subject was calculated. ROC curve analysis was performed for the API scores of ADNI-2 subjects.

ROC curve analyses were performed using MedCalc for Windows, version 18.2 (MedCalc Software, Ostend, Belgium), and all other analyses were performed using SPSS software, version 22 (IBM Corp., Armonk, NY, United States). The level of statistical significance was set as a two-tailed *p* < 0.05.

### Ethics Approval

This study was approved by the Institutional Review Boards of Seoul National University Hospital (IRB No: C-1401-027-547) and SNU-SMG Boramae Center (IRB No: 26-2015-60), Seoul, South Korea, and was conducted in accordance with the recommendations of the current version of the Declaration of Helsinki. All subjects or their legal representatives gave written and informed consent.

### Data Availability

The raw data supporting the conclusions of this manuscript will be made available by the authors, without undue reservation, to any qualified researcher.

## Results

### Participants

The Characteristics of the KBASE and ADNI-2 subjects included in this study are summarized in **Table 1**.

**Table 1:** Subject characteristics from the development and validation datasets.

Variables	KBASE	ADNI-2	T or χ^2^	*p*-value
N	176	476		
Age	73.2 ± 7.4	72.5 ± 7.7	0.97	0.332
Sex, female	117 (66.5)	208 (43.7)	26.67	<0.001
Years of education	9.6 ± 5.1	16.2 ± 2.6	−21.39	<0.001
Diagnosis, MCI, N (%)	107 (60.8)	332 (69.7)	4.68	0.030
Global CDR	0.8 ± 0.3	0.6 ± 0.2	9.31	<0.001
CDR-SB	2.9 ± 2.0	2.4 ± 1.8	2.73	0.006
MMSE, raw score	20.1 ± 4.6	26.5 ± 2.9	−17.03	<0.001
Amyloid positive, N (%)	100 (56.8)	325 (68.3)	7.44	0.006
APOE4 positive, N (%)	77 (43.8)	267 (56.1)	7.85	0.005

*KBASE, Korean Brain Aging Study for Early diagnosis and prediction of Alzheimer’s disease; ADNI, Alzheimer’s Disease Neuroimaging Initiative; df, degree of freedom; MCI, mild cognitive impairment; CDR, clinical dementia rating; CDR-SB, clinical dementia rating sum of box; MMSE, mini-mental state examination; APOE, apolipoprotein. Data for continuous variables are presented as mean ± SD and analyzed using student t-test with T-values presented in the table. Categorical variables are presented as N (%) and analyzed using a χ^2^ test with the χ^2^ values presented in the table.*

### Amyloid Prediction Model Developed From the KBASE Dataset

Through multivariate logistic regression analysis using the forward LR method, age, sex, years of education, history of hypertension, APOE4 positivity, and raw WLR score were selected for the final amyloid prediction model (**Table 2**). API was defined as the probability for a subject to be amyloid positive, which can be calculated from the logit value derived from the final multivariate logistic regression model for each subject. In the model, age and years of education were continuous variables. Sex was coded 0 for female and 1 for male, APOE 4 positivity was coded 0 for APOE4 non-carriers and 1 for APOE4 carriers, and a history of hypertension was coded 0 for the absence of hypertension history and 1 for the presence of hypertension history.

**Table 2:** The results of multivariate logistic regression analysis for the final prediction model for cerebral amyloid positivity.

Predictive variable	Beta	Wald Value	OR (95% CI)	*p*-value
Age	−0.025	0.81	0.98 (0.92 to 1.03)	0.370
Sex	−0.549	1.23	0.58 (0.22 to 1.53)	0.268
Years of education	0.141	8.87	1.15 (1.05 to 1.26)	0.003
APOE4 positivity	2.622	33.51	13.77 (5.67 to 33.44)	<0.001
History of hypertension	−1.400	10.76	0.25 (0.11 to 0.57)	0.001
WLR score	−0.440	13.07	0.64 (0.51 to 0.82)	<0.001
Intercept	1.551			

*OR, odds ratio; CI, confidence interval; APOE, apolipoprotein; WLR, word list recall. The result of the multivariate logistic regression analysis is shown with odds ratio and Wald value for each predictive variable.*

$$\begin{array}{l} {\mathrm{Logit}}_{\mathrm{case}}=\beta_{0}+\beta_{1}(\mathrm{age})+\beta_{2}\;(\mathrm{sex})+\beta_{3}(\mathrm{years of education})\\ \;+\beta_{4}(\mathrm{APOE4 positivity})+\beta_{5}(\mathrm{history of hypertension})\\ \;+\beta_{6}(\mathrm{raw score of word list recall test})\\ \;(\beta_{0}=1.551,{\;\beta }_{1}=-0.025,{\;\beta }_{2}=-0.549,{\;\beta }_{3}=0.141,\\ \;\beta_{4}=2.622,{\;\beta }_{5}=-1.400,{\;\beta }_{6}=-0.440)\\ \;{\mathrm{API}}_{\mathrm{case}}=\left[\exp\left({\mathrm{Logit}}_{\mathrm{case}}\right)\right]/[1+\exp\left({\mathrm{Logit}}_{\mathrm{case}}\right)] \end{array}$$

**FIGURE 1:**
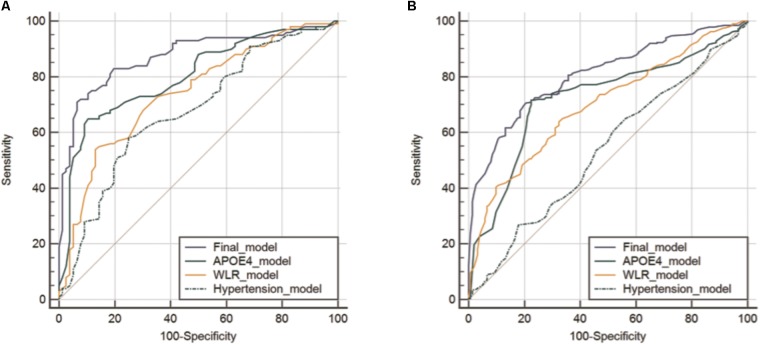
Receiver operating characteristic curves for the final and three simpler prediction models for cerebral amyloid positivity in KBASE **(A)** and ADNI-2 **(B)** datasets. Abbreviations: KBASE, Korean Brain Aging Study for Early diagnosis and prediction of Alzheimer’s disease; ADNI, Alzheimer’s Disease Neuroimaging Initiative; APOE, apolipoprotein; WLR, word list recall.

The AUC of the ROC curve for the developed model was 0.873 (95% confidence interval (CI) = 0.815 to 0.918) (**Table 3** and **Figure 1A**). **Table 4** shows the sensitivity, specificity, PPV, NPV, and accuracy at various cutoff points of the API. When 0.5 was used as a cutoff point (i.e., 50% probability for a subject to be amyloid positive), the sensitivity, specificity, PPV, NPV, and accuracy were 83.0, 80.3, 84.7, 78.2, and 81.8%, respectively. To compare with a simpler model, we also made three logistic regression models for amyloid prediction that included age, sex, years of education, and one of the three other variables in the full model (APOE4 positivity, WLR score, and history of hypertension) (**Table 5**). The AUC was 0.801 (95% CI = 0.734 to 0.857) for the APOE4 only model, 0.740 (95% CI = 0.669 to 0.803) for the WLR only model, and 0.675 (95% CI = 0.601 to 0.744) for the hypertension only model. Statistical comparison (DeLong et al., 1988) of the AUC between the full model and each of the simpler models showed that the full model had a significantly larger AUC than any simpler model (**Table 6**).

**Table 3:** The AUC values of ROC curves for the final and three simpler prediction models for cerebral amyloid positivity.

Pair	KBASE (*N* = 176)	ADNI-2 (*N* = 476)
Final model	0.873 (0.815 to 0.918)	0.808 (0.769 to 0.842)
APOE4 only model	0.801 (0.734 to 0.857)	0.723 (0.680 to 0.763)
WLR only model	0.740 (0.669 to 0.803)	0.697 (0.653 to 0.738)
Hypertension only model	0.675 (0.601 to 0.744)	0.535 (0.489 to 0.581)

*AUC, area under the curve; ROC, receiver operating characteristic; KBASE, Korean Brain Aging Study for Early diagnosis and prediction of Alzheimer’s disease; ADNI, Alzheimer’s Disease Neuroimaging Initiative; APOE, apolipoprotein; CI, confidence interval; WLR, word list recall. The AUC value for each model is presented with the 95% CI.*

**Table 4:** Sensitivity, specificity, positive, and negative predictive value, and accuracy at various cutoff points of API in the KBASE dataset (*N* = 176).

Cutoff for API	Sensitivity	Specificity	PPV	NPV	Accuracy
≥0.10	98.0	11.8	59.4	81.8	60.8
≥0.20	94.0	29.0	63.5	78.6	65.9
≥0.30	93.0	51.3	71.5	84.8	75.0
≥0.40	88.0	63.2	75.9	80.0	77.3
≥0.50	83.0	80.3	84.7	78.2	81.8
≥0.60	75.0	86.8	88.2	72.5	80.1
≥0.70	71.0	93.4	93.4	71.0	80.7
≥0.80	58.0	87.7	93.6	63.2	73.9
≥0.90	42.0	98.7	97.7	56.4	66.5

*API, amyloid prediction index; KBASE, Korean Brain Aging Study for Early diagnosis and prediction of Alzheimer’s disease; PPV, positive predictive value; NPV, negative predictive value. The results of exploration on how the sensitivity, specificity, positive predictive value (PPV), negative predictive value (NPV), and accuracy value change as the API increases from 0.1 to 0.9 by 0.1 are presented as percentage values.*

**Table 5:** The results of multivariate logistic regression analysis for simpler prediction models for cerebral amyloid positivity.

Predictive variable	Beta	Walds	OR (95% CI)	*p*-value
**APOE4 only model**				
Age	−0.027	1.19	0.97 (0.93 to 1.02)	0.275
Sex	−0.165	0.15	0.85 (0.36 to 1.98)	0.703
Years of education	0.080	3.83	1.08 (1.00 to 1.17)	0.050
APOE4 positivity	2.42	37.48	11.27 (5.19 to 24.46)	<0.001
Intercept	0.659			
**WLR only model**				
Age	−0.052	4.59	0.95 (0.91 to 1.00)	0.032
Sex	−0.397	0.95	0.67 (0.30 to 1.50)	0.672
Years of education	0.132	10.77	1.14 (1.06 to 1.24)	0.001
WLR	−0.432	18.14	0.65 (0.53 to 0.79)	<0.001
Intercept	3.84			
**Hypertension only model**				
Age	−0.022	0.95	0.98 (0.94 to 1.02)	0.330
Sex	−0.314	0.66	0.73 (0.34 to 1.56)	0.418
Years of education	0.086	5.60	1.09 (1.02 to 1.17)	0.018
Hypertension	−0.915	7.83	0.40 (0.21 to 0.76)	0.005
Intercept	1.647			

*OR, odds ratio; CI, confidence interval; APOE, apolipoprotein; WLR, word list recall. The result of multivariate logistic regression analysis is shown with odds ratio and Wald value for each predictive variable.*

**Table 6:** The pairwise comparison of receiver operating characteristic curves for the final and three simpler prediction models for cerebral amyloid positivity.

Pair	KBASE	ADNI-2
**Final model vs. APOE4 only model**		
Difference between areas (95% CI)	0.073 (0.030 to 0.115)	0.085 (0.050 to 0.120)
Z	3.33	4.79
*p*-value	<0.001	<0.001
**Final model vs. WLR only model**		
Difference between areas (95% CI)	0.133 (0.068 to 0.199)	0.111 (0.070 to 0.152)
Z	3.99	5.29
*p*-value	<0.001	<0.001
**Final model vs. Hypertension only model**		
Difference between areas (95% CI)	0.198 (0.117 to 0.279)	0.272 (0.195 to 0.350)
Z	4.79	6.90
*p*-value	<0.001	<0.001

*KBASE, Korean Brain Aging Study for Early diagnosis and prediction of Alzheimer’s disease; ADNI, Alzheimer’s Disease Neuroimaging Initiative; APOE, apolipoprotein; CI, confidence interval; WLR, word list recall. The results of pairwise comparison between prediction models using DeLong’s method are presented with z statistics and p-values.*

### External Validation of the Amyloid Prediction Model Using the ADNI-2 Dataset

The final amyloid prediction model developed using the KBASE dataset was externally validated using ADNI-2 data. The ADAS-cog delayed recall test scores included in the ADNI-2 assessment were used for the WLR score because the two tests have equivalent procedures, with an identical word list span and learning trials and a similar retention interval (Zhao et al., 2012). Since the ADAS-cog delayed recall score reflects the number of wrong answers while the WLR score counts the number of right answers, 10 minus the raw ADAS-cog delayed recall test score was used in the model. With regard to the other variables in the model, the same variables available in the ADNI-2 dataset were used. For the ADNI-2 data, the AUC of ROC curve was 0.808 (95% CI = 0.769 to 0.842) (**Table 3** and **Figure 1B**). Similar to the KBASE dataset, we also made three simpler logistic regression models that included age, sex, years of education, and one of the three other variables in the full model (APOE4 positivity, ADAS-cog delayed recall score, and history of hypertension) for the ADNI-2 dataset. The AUC was 0.723 (95% CI = 0.680 to 0.763) for the APOE4 model, 0.697 (95% CI = 0.653 to 0.738) for the WLR model, and 0.535 (95% CI = 0.489 to 0.581) for the hypertension model. The AUC of the full model was significantly greater than the simpler models.

## Discussion

The API, that is derived from commonly available variables in memory clinic practice, showed excellent capability (AUC 0.873) in screening cognitively impaired individuals with cerebral amyloid positivity. The results of external validation using data from an independent cohort with different characteristics were also satisfactory (AUC 0.808) and supported general applicability of the API.

Several previous studies have proposed prediction models for cerebral amyloid positivity. Bahar-Fuchs et al. focused on neuropsychological tests as potential predictive variables for cerebral amyloid positivity, and reported that delayed recall tests were the best predictors in 45 MCI patients referred from memory clinics (Bahar-Fuchs et al., 2013). After adjusting for confounding variables, the AUC for the ROC curve for each recall test was around 0.77∼0.86. However, the small sample size and lack of external validation limited the value of this model. Some excellent blood biomarker-based amyloid prediction models have also been reported (Burnham et al., 2014; Ovod et al., 2017; Park et al., 2017; Nakamura et al., 2018). Unfortunately, blood biomarkers are not yet readily available in clinical practice or clinical trials since they need further standardization, wide incorporation of the method, large-scale testing for normalization, and establishment of assay equipment. Haghighi et al. developed prediction models for cerebral amyloid positivity in 218 non-demented (168 MCI and 50 cognitively normal) subjects from the ADNI cohort (Haghighi et al., 2015). They proposed three models: one with neuropsychological variables, one with blood-based biomarkers, and the third with both variables. These models had AUCs of 0.76, 0.74, and 0.87, respectively. However, the blood-based biomarkers included in the second and third models included prostatic acid phosphatase, transthyretin, matrix metalloproteinase-10, and myoglobin, which are not usually available in general memory clinic practice settings. In addition, the models were not validated using an independent dataset. In contrast, the API from the current study can be easily applied to identify patients with a high probability of amyloid deposition in both clinical practice and therapeutic trials without further requirements. Since most of the recent AD clinical trials target amyloid positive patients who meet the NIA-AA criteria for MCI or AD dementia with a CDR 0.5 or 1, our API model may be useful for screening candidates for those trials, thereby reducing screening failure rate.

APOE4 positivity, history of hypertension, and WLR score were included in the final amyloid prediction model. The association between APOE4 status and amyloid positivity has been repeatedly reported in previous studies (Jack et al., 2015b; Jansen et al., 2015; Ossenkoppele et al., 2015; Gottesman et al., 2016). Similar to our results, these studies showed APOE4 positive MCI or AD dementia individuals were more likely to be amyloid positive. The scores of verbal delayed recall tests were also reported to be a reliable predictor for amyloid positivity in some previous studies (Bahar-Fuchs et al., 2013; Haghighi et al., 2015). History of hypertension was negatively correlated with amyloid positivity, and cognitively impaired individuals with a history of hypertension were less likely to be amyloid positive than those without a history of hypertension. Since chronic hypertension can reduce brain reserves through overall or regional brain atrophy (Wiseman et al., 2004; Glodzik et al., 2012; Beauchet et al., 2013; Power et al., 2016; Vemuri et al., 2017), individuals with a history of hypertension are more likely to be cognitively impaired with a relatively lower pathological burden, such as amyloid accumulation. Although APOE4, history of hypertension, and WLR score were each significantly related to cerebral amyloid positivity, the final model with all the three variables showed superior screening ability compared to the simper models with only one variable.

Of the demographic variables, years of education were positively associated with higher probability for being amyloid positive in our model. Since education is a well-known proxy for cognitive reserve (Meng and D’Arcy, 2012), highly educated individuals may endure more cerebral amyloid burden, as compared to those with lower educational levels (Roe et al., 2008; Rentz et al., 2010). Although age and sex are closely associated with AD pathophysiology (Gao et al., 1998; Jack et al., 2015b; Gottesman et al., 2016), they were not independently related to amyloid positivity in our study.

A few precautions should be mentioned for the model using the API. First, the performance of the prediction model or API was not sufficient to be used as a confirmatory tool for amyloid positivity and should be used only for screening purposes. Second, although we externally validated the model using data from individuals of different ethnic or cultural backgrounds, further validation is still needed for larger populations including individuals with various physical or psychiatric co-morbid conditions in order to broaden the model’s applicability.

## Conclusion

We developed a cerebral amyloid prediction model with variables that are commonly available in memory clinic practice. The prediction model proved to have excellent screening accuracy for amyloid positivity among cognitively impaired individuals, including MCI and mild AD dementia, through both internal and external validation. The API derived from the model can be useful for screening candidates with a high probability of amyloid deposition in therapeutic trials for early AD, as well as in clinical practice.

## Author Contributions

JL and DL designed the study, acquired and interpreted the data, and were major contributors to the writing of the manuscript and critically revising the manuscript for intellectual content. MB, DY, BS, SJ, YL, J-YL, YK, and Y-SL acquired and analyzed the data and helped to draft the manuscript. DL served as the principal investigator and supervised the study. All authors read and approved the final manuscript.

## Conflict of Interest Statement

The authors declare that the research was conducted in the absence of any commercial or financial relationships that could be construed as a potential conflict of interest.

## Footnotes

1 adni.loni.usc.edu

2 http://surfer.nmr.mgh.harvard.edu/
